# Efficacy and safety of chemotherapy plus immunotherapy in unresectable stage III–IV non-small cell lung cancer, with analysis of the additional role of anti-angiogenic agents

**DOI:** 10.3389/fonc.2026.1897228

**Published:** 2026-08-31

**Authors:** Xifen Qin, Zhiguo Wang, Hongbin Zhu

**Affiliations:** 1 The Fourth Affiliated Hospital of Anhui Medical University, Hefei, China; 2 Department of Respiratory and Critical Care Medicine, Anqing 116 Hospital, Anqing, China

**Keywords:** antiangiogenic therapy, chemotherapy, immunotherapy, NSCLC, real-world

## Abstract

**Purpose:**

Chemo-immunotherapy has demonstrated significant survival benefits in non-small cell lung cancer. However, real-world data on its effectiveness and safety in patients with unresectable stage III–IV disease remain insufficient. Furthermore, whether the addition of anti-angiogenic agents provides additional benefit beyond chemo-immunotherapy remains unclear. This study aimed to evaluate the real-world effectiveness and safety of adding immune checkpoint inhibitors to chemotherapy, and to further explore the added value of anti-angiogenic therapy.

**Methods:**

A total of 260 patients with unresectable stage III/IV NSCLC treated at two centers (2018–2025) were enrolled. Patients were divided into a chemotherapy-based immunotherapy group (n=162) and a non-immunotherapy chemotherapy group (n=98); the former was further stratified by the addition of anti-angiogenic therapy (n=71) or not (n=91). To address baseline imbalances between groups, propensity score matching (PSM) (1:1 nearest-neighbor, caliper=0.2 SD) was performed using key covariates. Progression-free survival and overall survival were assessed using Kaplan-Meier analysis and Cox regression. Adverse events were graded per CTCAE 5.0.

**Results:**

Chemotherapy-based immunotherapy significantly prolonged median PFS (9.0 vs 6.8 months, P<0.001) and OS (22 vs 14.2 months, P = 0.028) compared with non-immunotherapy chemotherapy regimens. This regimen was independently associated with improved PFS (HR = 0.56, P<0.001) and OS (HR = 0.65, P = 0.018). Conversely, ECOG PS 2 was a strong independent predictor of worse prognosis (PFS: HR = 6.45; OS: HR = 5.50, both P<0.001). Among recipients of chemotherapy‑based immunotherapy, adding anti-angiogenic therapy further improved PFS (9.1 vs 6.8 months, P = 0.044; HR = 0.69, P = 0.060), with no significant increase in adverse events (P > 0.05).

**Conclusions:**

Chemoimmunotherapy significantly improved PFS and OS in unresectable stage III/IV NSCLC. The addition of anti-angiogenic agents to this backbone further prolonged PFS without increasing adverse events.

## 1 Introduction

Lung cancer is one of the most common malignant tumors with the highest morbidity and mortality rates globally. Globally, lung cancer accounted for approximately 2.5 million new diagnoses and 1.8 million deaths in 2022, underscoring its status as a leading cause of cancer-related mortality worldwide (1). Among these, non-small cell lung cancer (NSCLC) accounts for approximately 85% of cases (2). Most patients are already in the middle or advanced stages at diagnosis, missing the opportunity for radical surgery (3, 4). Therefore, pharmacotherapy has become the primary treatment modality.

Traditional chemotherapy is the classic treatment regimen for middle- and advanced-stage lung cancer. Platinum-based drugs, combined with agents such as pemetrexed and paclitaxel, can effectively inhibit tumor cell proliferation and prolong patient survival. However, this approach faces an efficacy bottleneck because chemotherapeutic agents lack tumor specificity, making them prone to causing damage to normal tissues and triggering severe adverse reactions such as myelosuppression and gastrointestinal issues, which significantly impair patients’ quality of life (5–8). With the rapid advancement of medicine, immunotherapy and anti-angiogenic therapy have gradually emerged as new directions in the field of lung cancer treatment, breaking through the limitations of traditional chemotherapy.

Immunotherapy, represented by programmed death-1 (PD-1) and programmed death-ligand 1 (PD-L1) inhibitors, works by activating the body’s own immune system to recognize and eliminate tumor cells (9). It offers advantages such as durable efficacy and relatively mild adverse reactions (10, 11), making it an important option for first-line, second-line, and later-line treatment of advanced NSCLC. Anti-angiogenic therapy, on the other hand, inhibits tumor angiogenesis, thereby blocking the nutrient supply and metastatic pathways of tumors (12). Administered as a monotherapy or in combination with chemotherapy or immunotherapy, it can improve the prognosis of patients with advanced lung cancer. Drugs such as bevacizumab and anlotinib have been widely used in clinical practice.

In recent years, the application of immune checkpoint inhibitors (ICIs) in the treatment of advanced NSCLC has been proven to significantly improve the prognosis (13, 14) and has become an important treatment option for these patients. However, compared with immunotherapy, the synergistic therapy strategy of immunotherapy combined with anti-angiogenic drugs has been proven to have a theoretical basis for enhancing the anti-tumor effect in mechanism, and some clinical trials have shown its clinical value (15–19). At present, chemotherapy, immunotherapy, and anti-angiogenesis therapy play an important role in the treatment of lung cancer. But in the real world, different patients may choose different treatment options due to their age, physical status, underlying diseases, economic conditions, and treatment willingness. Despite the substantial survival benefits of chemo-immunotherapy established in landmark randomized controlled trials for advanced NSCLC, the generalizability of these findings to the broader, unselected real-world population remains incompletely defined.

Based on this, this study aims to retrospectively evaluate the efficacy and safety of adding immune checkpoint inhibitors to chemotherapy in patients with unresectable stage III–IV NSCLC, and to further assess whether the addition of anti-angiogenic agents confers additional clinical benefit. It also seeks to analyze the key factors influencing efficacy and treatment-related adverse events, thereby providing evidence-based medical evidence to assist clinicians in developing personalized treatment plans and optimizing therapeutic strategies for lung cancer, ultimately improving both the survival and quality of life of patients with lung cancer.

## 2 Materials and methods

### 2.1 Clinical data

**Figure 1:**
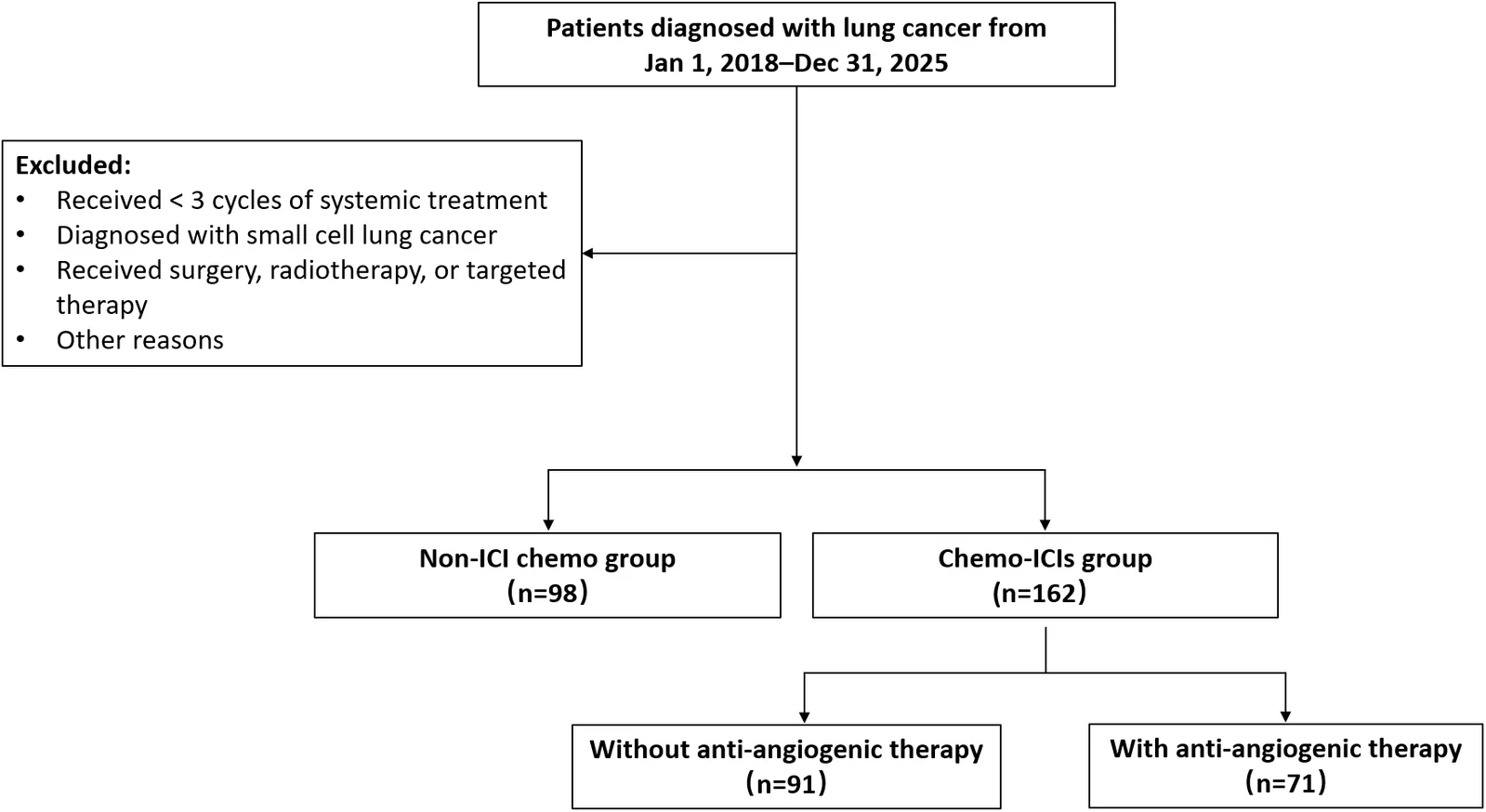
Flowchart of patient enrollment and grouping.

Patients with NSCLC were collected from the Fourth Affiliated Hospital of Anhui Medical University and the Anqing 116 Hospital from January 2018 to December 2025. Inclusion criteria: ① Histologically or cytologically confirmed unresectable stage III–IV non-small cell lung cancer (NSCLC), with clear pathological typing. Unresectability was defined as technically unresectable (e.g., bulky N2 disease, mediastinal infiltration of vital structures) or medically inoperable (e.g., advanced age, severe comorbidities, or ECOG PS ≥2) as determined by multidisciplinary team evaluation. Importantly, patients assessed as “potentially resectable” with the intent of conversion surgery following neoadjuvant therapy were strictly excluded from this cohort; ② Baseline imaging (CT/PET-CT) confirmed at least one measurable lesion (RECIST 1.1); ③ Receiving the target treatment regimen and completing at least 3 cycles of systemic treatment; ④ Complete clinicopathological data and follow-up data were available. Exclusion criteria: ① Having received other anti-tumor treatment at the same time (such as targeted therapy, radiotherapy, surgery, etc.); ② Combined with other primary tumors within 5 years, and severe heart, liver, kidney, and other important organ dysfunction; ③ Concurrent active infection or immune disease; ④ Key clinical data were missing. After screening, a total of 260 patients were included in the study (**Figure 1**). They were divided into 2 groups based on treatment regimen: the chemotherapy plus immune checkpoint inhibitors group (hereafter referred to as the chemo-ICIs group, n = 162), which comprised patients receiving chemotherapy plus ICIs with or without anti-angiogenic agents, and the chemotherapy without immune checkpoint inhibitors group (hereafter referred to as the non-ICI chemo group, n = 98), which comprised patients receiving chemotherapy without ICIs with or without anti-angiogenic agents. Within the chemo-ICIs group, patients were further divided into 2 subgroups: those receiving combined anti-angiogenic therapy (n=71) and those not receiving combined anti-angiogenic therapy (n=91). The detailed composition of treatment regimens in both the chemo-ICIs group and non-ICI chemo group, including the specific types of PD-1 inhibitors and anti-angiogenic agents (monoclonal antibodies vs. small-molecule TKIs), is summarized in **Supplementary Tables 1** and **2**.

### 2.2 Main outcome measures

The primary outcome measures were PFS (the time from the start of the treatment until the first documented progression of the disease, final follow-up, or any cause of death) and OS (the time between the start of the study treatment and death from any cause).

### 2.3 Evaluation of adverse events

Adverse events were coded according to the Medical Dictionary for Regulatory Activities (MedDRA) and classified by System Organ Class (SOC) and Preferred Term (PT), with severity graded per the Common Terminology Criteria for Adverse Events version 5.0 (CTCAE 5.0).

### 2.4 Statistical analysis

Statistical analyses were performed using SPSS (version 26.0) and R software (version 4.6). The measurement data conformed to the normal distribution and were expressed as “mean ± standard deviation”. T-test or analysis of variance was used for comparison between groups. The non-normally distributed data were expressed as “median (Q1, Q3)”, and the Mann-Whitney U test was used for comparison between groups. Enumeration data were expressed as “frequency (percentage)”, and the chi-square (χ2) test was used for comparison between groups. To minimize potential selection bias and confounding due to imbalances in baseline characteristics between groups, propensity score matching (PSM) was performed using R software with the MatchIt package. Propensity scores were estimated using a logistic regression model incorporating key covariates, including age, sex, smoking status, ECOG performance status, histology, and clinical stage. Patients were matched using a 1:1 nearest-neighbor matching algorithm without replacement, with a caliper of 0.2 times the standard deviation of the logit of the propensity score. Standardized mean differences (SMDs) were calculated to assess covariate balance between groups, and an SMD of <0.1 was considered indicative of adequate balance. To account for residual imbalances, any covariate with an SMD > 0.1 following PSM was entered into the multivariable Cox regression, ensuring robust estimation of treatment effects.

Survival curves were plotted by the Kaplan-Meier method, and the Log-rank test was used to make comparisons. Cox regression models were used to find the prognostic factors associated with PFS and OS. A value of P<0.05 was considered statistically significant. The safety analysis used nonparametric test to compare the AEs.

## 3 Results

### 3.1 Comparison of the non-ICI chemo group versus chemo-ICIs group

#### 3.1.1 Patient characteristics

According to the inclusion and exclusion criteria, a total of 260 patients with NSCLC were included. The median follow-up time for the overall cohort was 40 months (95% CI: 34.6 – 57.6). Among all eligible patients, the median age was 71 years, 88.1% were male, the median BMI was 21.61kg/m², 34.6% were smokers, 59.6% were squamous cell carcinoma, 41.9% were stage III according to the AJCC Version 9, 58.1% were stage IV according to the AJCC Version 9, and 95.8% had an Eastern Cooperative Oncology Group performance status (ECOG PS) of 0–1. **Table 1** summarizes the baseline characteristics of the two groups before and after propensity score matching (PSM). Before matching, significant differences were observed in histological type and clinical stage between groups (P < 0.05), while the remaining covariates, including age, gender, BMI, smoking history, and ECOG PS, were well balanced (all P > 0.05). To address these imbalances, PSM was performed incorporating the following covariates: age, gender, histological type, clinical stage, smoking status, ECOG PS, and BMI, using a 1:1 nearest-neighbor matching algorithm. After matching, all baseline characteristics became comparable between the two groups (all P > 0.05), indicating that the matching procedure effectively minimized selection bias.

**Table 1:** Baseline characteristics of patients stratified by treatment group (non-ICI chemo vs. chemo-ICIs) before and after propensity score matching.

Characteristic	Before PSM				After PSM			
	Non-ICI chemo	Chemo-ICIs	Difference^2^	P value	Non-ICI chemo	Chemo-ICIs	Difference^2^	P value
	N = 98^1^	N = 162^1^			N = 89^1^	N = 89^1^		
Gender			0.21	0.088			0	1.000
Female	16 (16.3%)	15 (9.3%)			11 (12.4%)	11 (12.4%)		
Male	82 (83.7%)	147 (90.7%)			78 (87.6%)	78 (87.6%)		
**age**	70 (65, 76)	72 (66, 76)	-0.09	0.303	69 ± 8.5	69 ± 9.2	0.02	0.873
**BMI**	22.0 ± 3.4	21.8 ± 2.9	0.09	0.491	21.7 (20.0, 23.5)	21.5 (19.0, 24.2)	0.11	0.510
Smoking status			0.04	0.772			0.27	0.074
Current or former smoker	35 (35.7%)	55 (34%)			33 (37.1%)	22 (24.7%)		
Never smoked	63 (64.3%)	107 (66%)			56 (62.9%)	67 (75.3%)		
Histology			0.53	<0.001			0.07	0.653
Squamous carcinoma	43 (43.9%)	112 (69.1%)			43 (48.3%)	46 (51.7%)		
Adenocarcinoma	55 (56.1%)	50 (30.9%)			46 (51.7%)	43 (48.3%)		
Stage			0.27	0.036			0.14	0.341
III	33 (33.7%)	76 (46.9%)			33 (37.1%)	27 (30.3%)		
IV	65 (66.3%)	86 (53.1%)			56 (62.9%)	62 (69.7%)		
ECOG PS			0.01	1.000^b^			0.21	0.364^b^
0-1	94 (95.9%)	155 (95.7%)			85 (95.5%)	88 (98.9%)		
2	4 (4.1%)	7 (4.3%)			4 (4.5%)	1 (1.1%)		

^1^ n (%); Median (Q1, Q3).

^2^ Standardized Mean Difference.

^b^ P-value was calculated using the Chi-square test with Yates’ continuity correction.

#### 3.1.2 Efficacy

**Figure 2:**
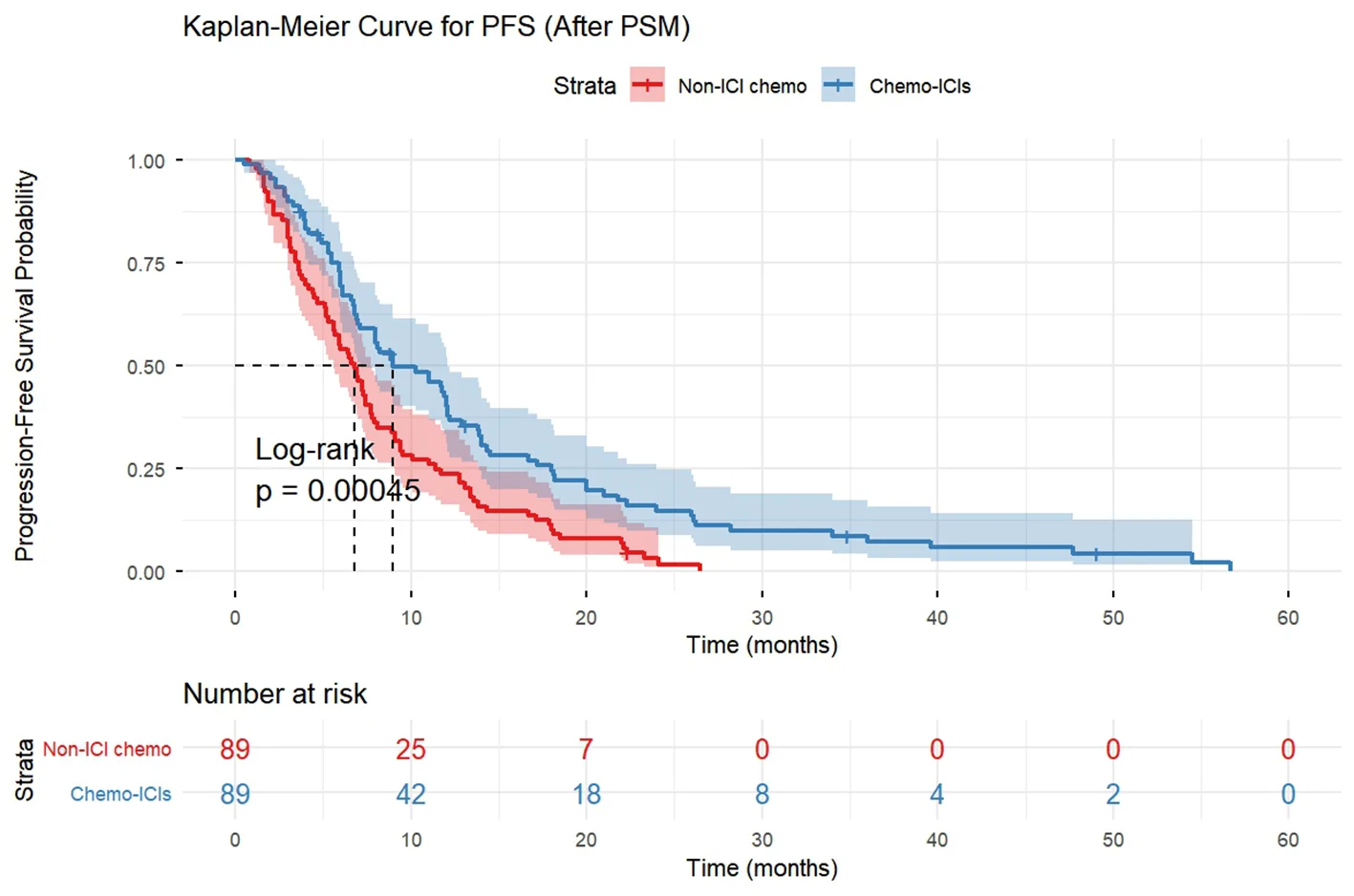
Kaplan-Meier curves of PFS for the chemo-ICIs group versus non-ICI chemo groups, with numbers at risk.

**Figure 3:**
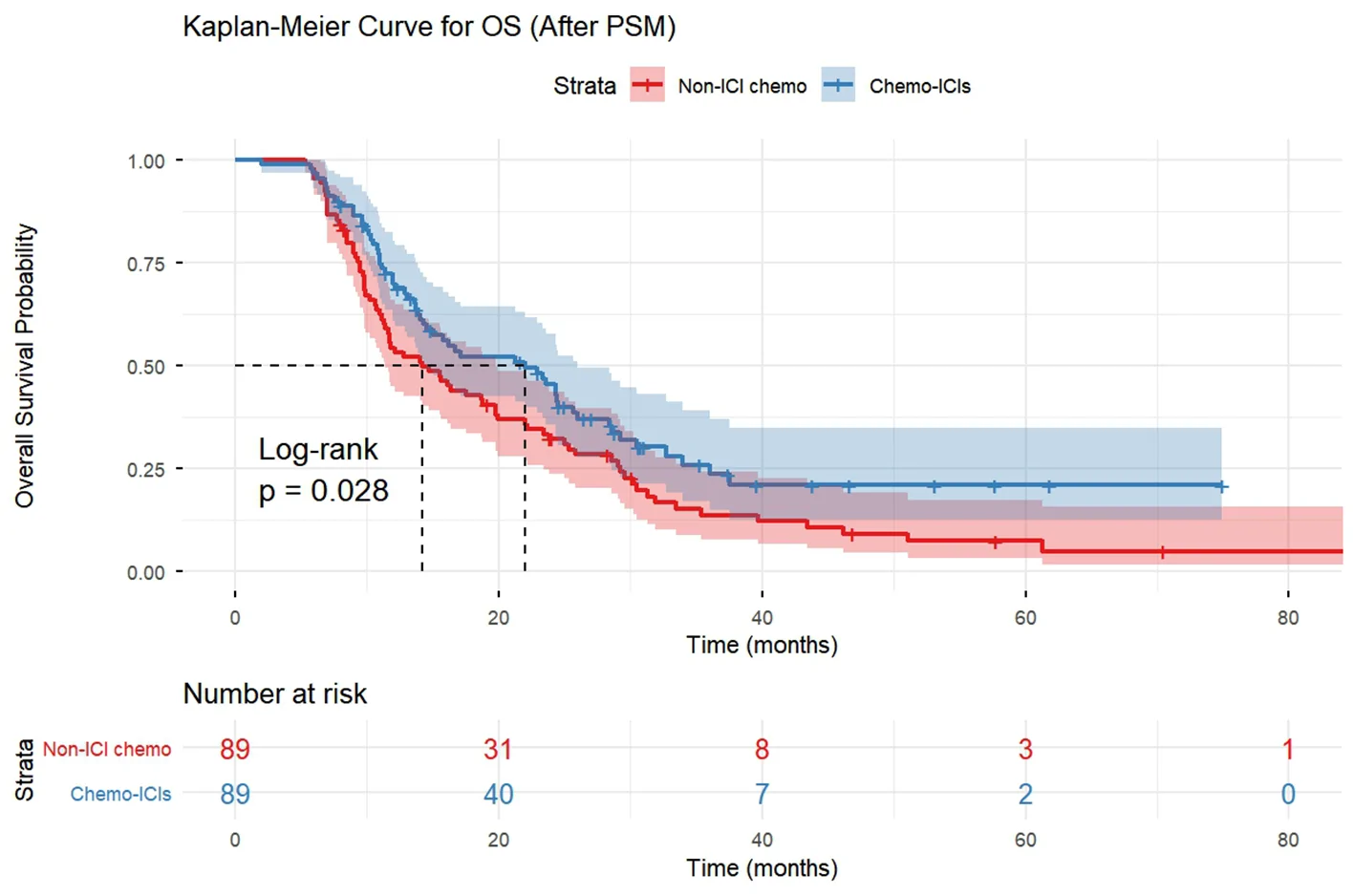
Kaplan-Meier curves of OS for the chemo-ICIs group versus non-ICI chemo groups, with numbers at risk.

After propensity score matching (PSM), Kaplan-Meier analysis was performed on the matched cohort (n = 178). The chemo-ICIs group had significantly longer median PFS than the non-ICI chemo group: 9.0 months (95% CI: 7.1–12.1) vs. 6.8 months (95% CI: 5.6–7.7) (P < 0.001). Similarly, the median OS was significantly improved in the chemo-ICIs group: 22.0 months (95% CI: 14.5–26.0) vs. 14.2 months (95% CI: 11.4–19.8) (P = 0.028). The corresponding survival curves, along with the number at risk tables, are presented in **Figures 2** and **3**.

#### 3.1.3 Analysis of prognostic factors

Multivariable Cox regression analysis was performed to identify independent prognostic factors for PFS and OS. For PFS, univariate analysis demonstrated that treatment regimen (chemo-ICIs group vs. non-ICI chemo group) and ECOG PS (2 vs. 0-1) were significantly associated with PFS (both P < 0.001). BMI and smoking status, which exhibited residual imbalance after PSM (SMD > 0.1), were included to minimize residual confounding through double adjustment. The remaining covariates—age, histological type, clinical stage, and ECOG PS—were forcibly entered into the model based on their established prognostic relevance for NSCLC. After adjustment, the chemotherapy plus immune checkpoint inhibitors regimen remained an independent protective factor for PFS (HR = 0.56, 95% CI: 0.40–0.77, P < 0.001), while ECOG PS 2 was identified as an independent adverse prognostic factor (HR = 6.45, 95% CI: 2.48–16.8, P < 0.001) (**Table 2**).

**Table 2:** Univariate and multivariate Cox regression analyses of PFS in the chemo-ICIs group versus non-ICI chemo groups after PSM.

Characteristic	Univariate				Multivariate		
	N	HR	95% CI	p-value	HR	95% CI	p-value
Treatment (Chemo-ICIs vs Non-ICI chemo)	178	0.58	0.42, 0.79	<0.001	0.56	0.40, 0.77	<0.001
Gender (Male vs Female)	178	1.04	0.66, 1.65	0.864			
Age (years)	178	1.01	0.99, 1.02	0.546	1.01	0.99, 1.03	0.383
BMI (kg/m2)	178	1.00	0.96, 1.05	0.849	1.02	0.97, 1.07	0.416
Smoking (Yes vs No)	178	1.01	0.72, 1.40	0.974	0.98	0.70, 1.38	0.920
Histology (Adenocarcinoma vs Squamous carcinoma)	178	0.95	0.70, 1.29	0.756	0.84	0.60, 1.17	0.311
Stage (IV vs III)	178	0.88	0.64, 1.21	0.417	0.94	0.67, 1.33	0.742
ECOG PS (2 vs 0-1)	178	5.55	2.22, 13.9	<0.001	6.45	2.48, 16.8	<0.001

CI, Confidence Interval, HR, Hazard Ratio.

For OS, univariate analysis identified treatment regimen (P = 0.028) and ECOG PS (P < 0.001) as candidate predictors (P < 0.10), which were subsequently entered into the multivariable model alongside the covariates adjusted in the PFS analysis. On multivariable analysis, the chemotherapy plus immune checkpoint inhibitors regimen remained independently associated with a significantly reduced risk of death (HR = 0.65, 95% CI: 0.46–0.93, P = 0.018). ECOG PS 2 was again confirmed as a strong independent adverse prognostic factor for OS (HR = 5.50, 95% CI: 2.08–14.5, P < 0.001). Although patients with stage IV disease showed a trend toward worse OS compared with stage III, the difference did not reach statistical significance (HR = 1.37, 95% CI: 0.92–2.04, P = 0.121). Other covariates were not significantly associated with OS in either univariate or multivariable analyses (all P > 0.05) (**Table 3**).

**Table 3:** Univariate and multivariate Cox regression analyses of OS in the chemo-ICIs group versus non-ICI chemo groups after PSM.

Characteristic	Univariate				Multivariate		
	N	HR	95% CI	p-value	HR	95% CI	p-value
Treatment (Chemo-ICIs vs Non-ICI chemo)	178	0.68	0.49, 0.96	0.028	0.65	0.46, 0.93	0.018
Gender (Male vs Female)	178	1.00	0.61, 1.65	0.986			
Age (years)	178	1.01	0.99, 1.03	0.191	1.01	0.99, 1.03	0.198
BMI (kg/m2)	178	0.97	0.92, 1.02	0.259	0.99	0.93, 1.04	0.616
Smoking (Yes vs No)	178	1.09	0.75, 1.57	0.651	1.09	0.74, 1.61	0.646
Histology (Adenocarcinoma vs Squamous carcinoma)	178	0.91	0.65, 1.27	0.583	0.76	0.52, 1.11	0.153
Stage (IV vs III)	178	1.20	0.83, 1.73	0.327	1.37	0.92, 2.04	0.121
ECOG PS (2 vs 0-1)	178	6.53	2.56, 16.7	<0.001	5.50	2.08, 14.5	<0.001

CI, Confidence Interval; HR, Hazard Ratio.

#### 3.1.4 Adverse reactions

As shown in **Table 4**, the overall incidence of any-grade AEs was 92.1% in the chemo-ICIs group versus 100% in the non-ICI chemo group (P = 0.025), with a higher proportion of grade 3–4 events in the latter (52.8% vs. 47.2%). The chemo-ICIs group was associated with significantly lower risks of myelosuppression (P = 0.007), nausea/vomiting (P = 0.003), and decreased appetite (P = 0.001), but higher risks of hypothyroidism (P = 0.036).

**Table 4:** Treatment-emergent adverse events by SOC, PT, and severity grade in the chemo-ICIs group vs. non−ICI chemo group.

System organ class (SOC) /	Chemo-ICIs (n=89)		Non-ICI chemo (n=89)		P value
Preferred term (PT)–n (%)	Grade 1–2 Grade 3–4		Grade 1–2 Grade 3–4		
**Any adverse event**	40 (44.9)	42 (47.2)	42 (47.2)	47 (52.8)	0.025^a^
Blood and lymphatic system	43 (48.3)	34 (38.2)	49 (55.1)	39 (43.8)	0.007
Neutropenia	18 (20.2)	16 (18.0)	26 (29.2)	14 (15.7)	0.38
Leukopenia	24 (27.0)	11 (12.4)	34 (38.2)	13 (14.6)	0.184
Anemia	51 (57.3)	23 (25.8)	55 (61.8)	32 (36.0)	0.003
Thrombocytopenia	33 (37.1)	6 (6.7)	47 (52.8)	8 (9.0)	0.055
Gastrointestinal disorders	4 (4.5)	0	17 (19.1)	0	0.003
Nausea	3 (3.4)	0	15 (16.9)	0	0.003
Vomiting	3 (3.4)	0	15 (16.9)	0	0.003
Hepatobiliary disorders	30 (33.7)	9 (10.1)	44 (49.4)	7 (7.9)	0.104
ALT increased	27 (30.3)	3 (3.4)	33 (37.1)	3 (3.4)	0.592^a^
AST increased	25 (28.1)	7 (7.9)	38 (42.7)	6 (6.7)	0.124
Blood bilirubin increased	5 (5.6)	3 (3.4)	8 (9.0)	1 (1.1)	0.498^a^
Renal and urinary disorders	18 (20.2)	3 (3.4)	23 (25.8)	0	0.173^a^
Blood creatinine increased	13 (14.6)	3 (3.4)	14 (15.7)	0	0.316^a^
Proteinuria	9 (10.1)	0	13 (14.6)	0	0.362
Endocrine disorders	12 (13.5)	0	4 (4.5)	0	0.036
Hypothyroidism					
Metabolism and nutrition disorders	56 (62.9)	9 (10.1)	69 (77.5)	11 (12.4)	0.015
Decreased appetite	15 (16.9)	1 (1.1)	35 (39.3)	0	0.001^a^
Hypercholesterolemia	29 (32.0)	1 (1.1)	28 (31.5)	0	0.873^a^
Hyperuricemia	28 (31.5)	7 (7.9)	38 (42.7)	9 (10.1)	0.195
Hypoalbuminemia	34 (38.2)	2 (2.2)	49 (55.1)	2 (2.2)	0.064^a^
Skin and subcutaneous tissue disorders	6 (6.7)	2 (2.2)	4 (4.5)	0	0.315^a^
Pruritus					
General disorders and administration site conditions	9 (10.1)	0	17 (19.1)	0	0.09
Asthenia					
Respiratory, thoracic and mediastinal disorders	2 (2.2)	0	0	0	0.497^a^
Immune-related pneumonia					

^a^ Fisher's exact test (two-sided).

In addition, we paid special attention to the occurrence of checkpoint inhibitor-associated pneumonitis (CIP), which is a serious adverse event of concern in immunotherapy with checkpoint inhibitors. In this study, only 2 cases (2.2%) of grade 1–2 CIP were observed in the chemo-ICIs group. All CIP cases were mild to moderate, and improved after treatment with glucocorticoids. No grade 3–4 or fatal pneumonia events occurred.

In conclusion, the two regimens exhibited distinct AE profiles, with chemoimmunotherapy showing a more favorable gastrointestinal and hematologic safety profile.

### 3.2 Comparison of combined anti-angiogenic therapy versus non-combined therapy

#### 3.2.1 Patient characteristics

In the overall chemotherapy plus ICIs cohort, the median age was 71.5 years, 90.7% were male, the mean BMI was 21.76 kg/m², 34% were smokers, 69.1% were squamous cell carcinoma, 46.9% were stage III according to the AJCC Version 9, and 95.7% had an ECOG PS of 0–1. Before PSM, the two groups were well balanced across most covariates, except for histology (P = 0.037). As shown in **Table 5**, the baseline characteristics of the chemo−ICIs cohort with versus without anti−angiogenic therapy were compared before and after PSM. After PSM, all covariates, including age, gender, BMI, smoking, stage, and ECOG PS, were comparable between groups (all P > 0.05, SMD < 0.1).

**Table 5:** Baseline characteristics of patients stratified by treatment group (without anti-angiogenic therapy vs. with anti-angiogenic therapy) before and after propensity score matching.

Characteristic	Before PSM				After PSM			
	Without anti-angiogenic therapy	With anti-angiogenic therapy	Difference^2^	P value	Without anti-angiogenic therapy	With anti-angiogenic therapy	Difference^2^	P value
	N = 91^1^	N = 71^1^			N = 63^1^	N = 63^1^		
Gender			0.21	0.185			0.06	1.000^b^
Female	6 (6.6%)	9 (12.7%)			5 (7.9%)	4 (6.3%)		
Male	85 (93.4%)	62 (87.3%)			58 (92.1%)	59 (93.7%)		
**age**	72 (66, 77)	71 (66, 76)	-0.02	0.839	73 (67, 77)	71 (67, 76)	0.04	0.723
**BMI**	21.7 ± 2.8	21.8 ± 3.1	-0.05	0.746	21.7 ± 2.6	21.8 ± 3.1	-0.04	0.816
Smoking status			0.01	0.972			0.03	0.855
Current or former smoker	31 (34.1%)	24 (33.8%)			25 (39.7%)	24 (38.1%)		
Never smoked	60(65.9%)	47 (66.2%)			38(60.3%)	39 (61.9%)		
Histology			0.33	0.037			0.1	0.563
Squamous carcinoma	69 (75.8%)	43 (60.6%)			45 (71.4%)	42 (66.7%)		
Adenocarcinoma	22 (24.2%)	28 (39.4%)			18 (28.6%)	21 (33.3%)		
Stage			0.17	0.294			0	1.000
III	46 (50.5%)	30 (42.3%)			29 (46%)	29 (46%)		
IV	45 (49.5%)	41 (57.7%)			34 (54%)	34 (54%)		
ECOG PS			0.01	1.000^b^			0.08	1.000^b^
0-1	87 (95.6%)	68 (95.8%)			60 (95.2%)	61 (96.8%)		
2	4 (4.4%)	3 (4.2%)			3 (4.8%)	2 (3.2%)		

^1^ n (%); Median (Q1, Q3).

^2^ Standardized Mean Difference

^b^ P-value was calculated using the Chi-square test with Yates’ continuity correction.

#### 3.2.2 Efficacy

**Figure 4:**
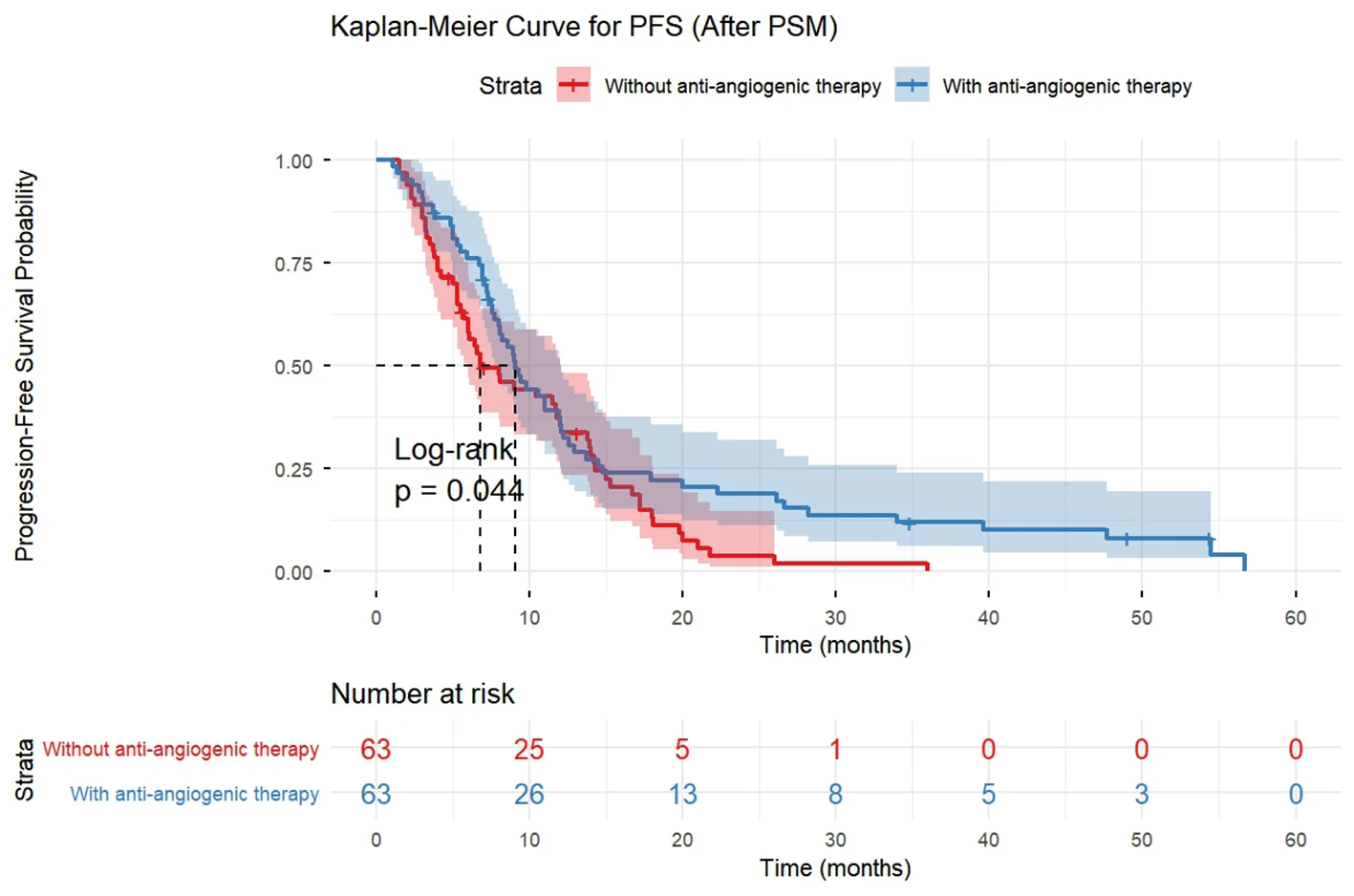
Kaplan-Meier curves of PFS for the without anti-angiogenic therapy versus with anti-angiogenic therapy, with numbers at risk.

**Figure 5:**
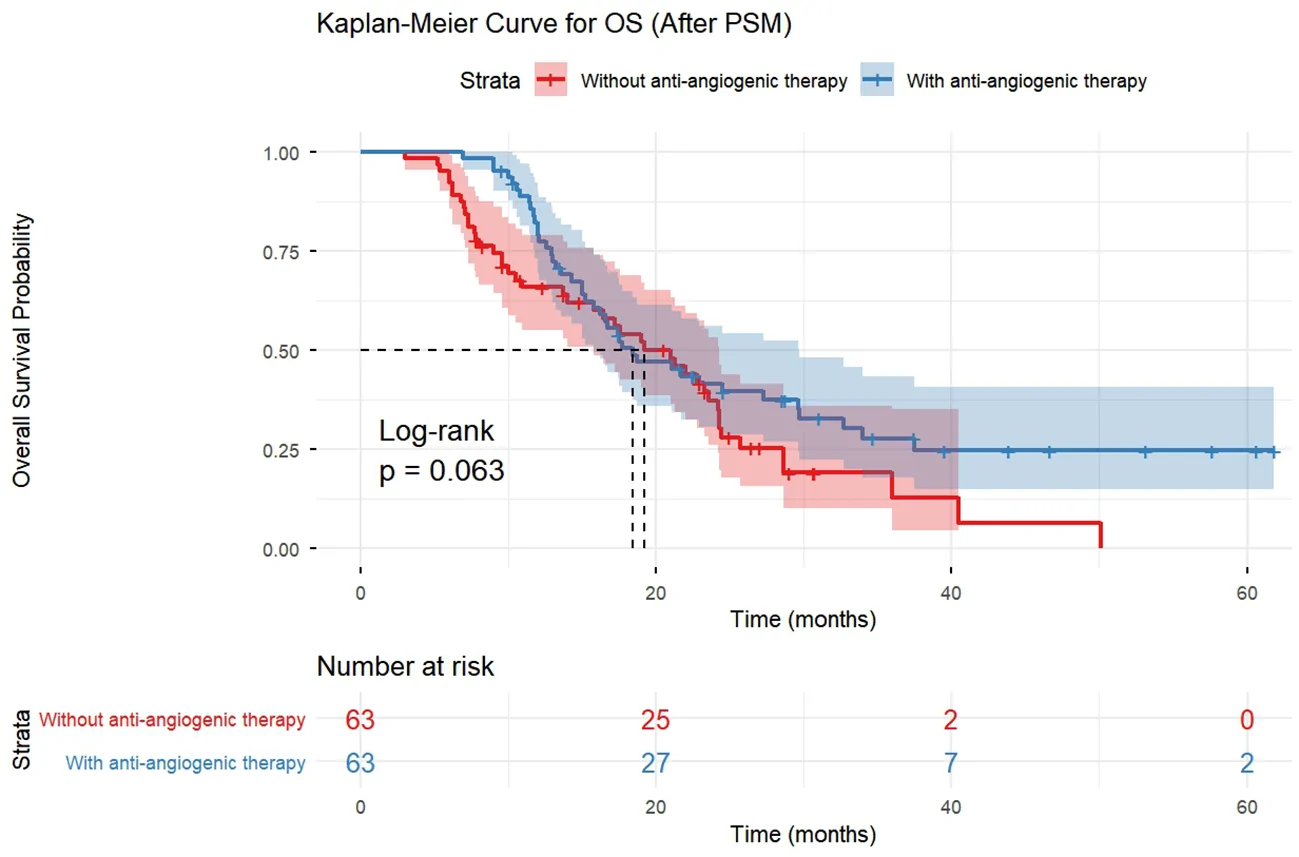
Kaplan-Meier curves of OS for the without anti-angiogenic therapy versus with anti-angiogenic therapy, with numbers at risk.

After propensity score matching, in subgroup analysis, the median PFS of patients with and without anti-angiogenic therapy was significantly different: 9.1 months (95% CI: 8.0–12.1) vs 6.8 months (95% CI: 6.0–12.0) (P = 0.044). The median OS was 18.4 months (95% CI: 15.8–29.7) for the combination group and 19.2 months (95% CI: 15.8–24.3) for the non-combination group, with no significant difference between the two groups (P = 0.063). (**Figures 4**, **5**)

#### 3.2.3 Analysis of prognostic factors

Univariate analysis showed that only the addition of anti−angiogenic therapy was significantly associated with PFS (HR = 0.68, 95% CI: 0.46–0.99, P = 0.044). However, in the multivariable analysis adjusting for age, histology, stage, and ECOG PS, the association did not retain statistical significance (HR = 0.69, 95% CI: 0.47–1.02, P = 0.060), suggesting a potential trend toward PFS benefit with the addition of anti−angiogenic agents that did not reach significance after adjustment. No other covariates were significantly associated with PFS in either univariate or multivariable analyses (all P > 0.05) (**Table 6**).

**Table 6:** Univariate and multivariate Cox regression analysis of PFS in with anti-angiogenic therapy vs. without anti-angiogenic therapy groups after PSM.

Characteristic	Univariate				Multivariate		
	N	HR	95% CI	p-value	HR	95% CI	p-value
Treatment (With anti-angiogenic therapy vs Without anti-angiogenic therapy)	126	0.68	0.46, 0.99	0.044	0.69	0.47, 1.02	0.060
Gender (Male vs Female)	126	1.09	0.55, 2.16	0.810			
Age (years)	126	1.02	0.99, 1.04	0.179	1.01	0.99, 1.04	0.324
BMI (kg/m2)	126	0.99	0.93, 1.05	0.784			
Smoking (Yes vs No)	126	1.04	0.71, 1.53	0.839			
Histology (Adenocarcinoma vs Squamous carcinoma)	126	0.85	0.57, 1.27	0.425	0.91	0.58, 1.43	0.686
Stage (IV vs III)	126	0.91	0.63, 1.33	0.633	0.95	0.64, 1.40	0.781
ECOG PS (2 vs 0-1)	126	1.46	0.54, 3.99	0.457	1.55	0.54, 4.44	0.418

CI, Confidence Interval; HR, Hazard Ratio.

For OS, neither the addition of anti−angiogenic therapy nor any of the other covariates—including gender, age, BMI, smoking, histology, stage, or ECOG PS—demonstrated a statistically significant association in univariate or multivariable analyses (all P > 0.05). Notably, ECOG PS 2 showed a trend toward worse OS in univariate analysis (HR = 2.68, 95% CI: 0.97–7.39, P = 0.057), which was attenuated in the multivariable model (HR = 2.25, 95% CI: 0.77–6.60, P = 0.140) (**Table 7**).

**Table 7:** Univariate and multivariate Cox regression analysis of OS in with anti-angiogenic therapy vs. without anti-angiogenic therapy groups after PSM.

Characteristic	Univariate				Multivariate		
	N	HR	95% CI	p-value	HR	95% CI	p-value
Treatment (With anti-angiogenic therapy vs Without anti-angiogenic therapy)	126	0.67	0.44, 1.02	0.064	0.66	0.43, 1.01	0.054
Gender (Male vs Female)	126	0.64	0.32, 1.28	0.209			
Age (years)	126	1.00	0.97, 1.02	0.957	1.00	0.98, 1.03	0.827
BMI (kg/m2)	126	0.96	0.89, 1.03	0.225			
Smoking (Yes vs No)	126	0.82	0.52, 1.29	0.381			
Histology (Adenocarcinoma vs Squamous carcinoma)	126	1.16	0.75, 1.81	0.502	1.03	0.63, 1.67	0.920
Stage (IV vs III)	126	1.38	0.89, 2.12	0.147	1.36	0.87, 2.15	0.180
ECOG PS (2 vs 0-1)	126	2.68	0.97, 7.39	0.057	2.25	0.77, 6.60	0.140

CI, Confidence Interval; HR, Hazard Ratio.

#### 3.2.4 Adverse reactions

No significant differences were observed in the incidence of AEs across any system organ class or preferred term between the two groups (all P > 0.05). In this study, no cases of CIP were observed in the combination group, whereas 1 case (1.6%) of grade 1–2 CIP occurred in the group without anti−angiogenic therapy. Overall, the incidence of CIP remained low across both groups. These findings suggest that the addition of anti−angiogenic agents to chemo−immunotherapy did not substantially increase the risk of treatment−emergent adverse events. (**Table 8**)

**Table 8:** Treatment-emergent adverse events by SOC, PT and severity grade in anti-angiogenic combination vs. non-anti-angiogenic patients.

System organ class (SOC)/Preferred term (PT)–n (%)	With anti-angiogenic therapy		Without anti-angiogenic therapy		P value
	(n=63)		(n=63)		
	Grade 1–2 Grade 3–4		Grade 1–2 Grade 3–4		
**Any adverse event**	33 (52.4)	28 (44.4)	32 (50.8)	27 (42.9)	0.828^a^
Blood and lymphatic system	36 (57.1)	23 (36.5)	36 (57.1)	20 (31.7)	0.598
Neutropenia	10 (15.9)	11 (17.5)	18 (28.6)	13 (20.6)	0.149
Leukopenia	17 (27.0)	9 (14.3)	27 (42.9)	6 (9.5)	0.165
Anemia	39 (61.9)	18 (28.6)	43 (68.3)	12 (19.0)	0.432
Thrombocytopenia	33 (52.4)	1 (1.6)	24 (38.1)	5 (7.9)	0.094^a^
Gastrointestinal disorders	5 (7.9)	0	2 (3.2)	0	0.440^a^
Nausea	4 (6.3)	0	2 (3.2)	0	0.680^a^
Vomiting	3 (4.8)	0	1 (1.6)	0	0.619^a^
Hepatobiliary disorders	24 (38.1)	5 (7.9)	27 (42.9)	6 (9.5)	0.772
ALT increased	21 (33.3)	2 (3.2)	18 (28.6)	2 (3.2)	0.885^a^
AST increased	21 (33.3)	4 (6.3)	21 (33.3)	5 (7.9)	1.000^a^
Blood bilirubin increased	3 (4.8)	2 (3.2)	6 (9.5)	2 (3.2)	0.677^a^
Renal and urinary disorders	11 (17.5)	3 (4.8)	16 (25.4)	0	0.156^a^
Blood creatinine increased	7 (11.1)	3 (4.8)	11 (17.5)	0	0.164^a^
Proteinuria	7 (11.1)	0	8 (12.7)	0	0.783
Endocrine disorders	9 (14.3)	0	6 (9.5)	0	0.409
Hypothyroidism					
Metabolism and nutrition disorders	43 (68.3)	8 (12.7)	36 (57.1)	9 (14.3)	0.391
Decreased appetite	15 (23.8)	0	15 (23.8)	1 (1.6)	1.000^a^
Hypercholesterolemia	19 (30.2)	0	15 (23.8)	1 (1.6)	0.547^a^
Hyperuricemia	18 (28.6)	6 (9.5)	16 (25.4)	7 (11.1)	0.902
Hypoalbuminemia	32 (50.8)	2 (3.2)	24 (38.1)	2 (3.2)	0.334^a^
Skin and subcutaneous tissue disorders	3 (4.8)	2 (3.2)	4 (6.3)	0	0.554^a^
Pruritus					
General disorders and administration site conditions	7 (11.1)	0	9 (14.3)	0	0.593
Asthenia					
Respiratory, thoracic and mediastinal disorders	0	0	1 (1.6)	0	1.000^a^
Immune-related pneumonia					

^a^ Fisher’s exact test (two-sided).

## 4 Discussion

Multiple large-scale randomized controlled trials (RCTs) have confirmed the effectiveness of chemo-immunotherapy for NSCLC, providing high-level evidence for the development of clinical guidelines. While RCTs offer high internal validity, their external validity is inherently limited. The strict inclusion and exclusion criteria of RCTs mean that the enrolled populations are often “ideal patients”, with favorable age, excellent performance status, few comorbidities, and high compliance. These “standardized patients” who meet clinical trial eligibility criteria represent only a fraction of the real-world population, whereas the complexity and diversity of real-world advanced NSCLC patients far exceed the spectrum covered by RCTs, with substantial differences in comorbidities, performance status, organ function reserve, financial affordability, and treatment tolerance. For instance, the standard of care for unresectable stage III NSCLC is concurrent chemoradiotherapy followed by consolidation durvalumab, as established by the landmark PACIFIC trial (20–22). However, the implementation of this standard regimen in the real-world setting faces multiple challenges. Some patients are physiologically ineligible due to advanced age, severe cardiopulmonary comorbidities, or poor performance status, and cannot tolerate the intensity of concurrent chemoradiotherapy or the subsequent 12-month consolidation immunotherapy. Furthermore, economic constraints, drug accessibility, and patient treatment preferences also prevent a substantial proportion of patients from receiving or accepting this standard regimen. Consequently, these patients ultimately receive chemotherapy-based systemic therapy as their primary palliative treatment in real-world clinical practice.

Our patient cohort comprehensively reflects the characteristics of this population often overlooked by clinical trials. In this real-world context, the primary objective of our study is to evaluate whether chemo-immunotherapy still confers survival benefits in a broader real-world population across various patient subgroups with different comorbidities, performance statuses, and clinical characteristics, thereby providing more practically actionable evidence to inform clinical decision-making. This is a clinical reality that RCT evidence alone cannot directly address and must be answered by real-world research. In addition to evaluating the efficacy of chemo-immunotherapy itself, our study further explores whether the addition of anti-angiogenic therapy to chemo-immunotherapy provides additional clinical benefit, a question that remains unanswered in the real-world context and warrants further investigation. Although this study does not yield novel biomarkers or mechanistic breakthroughs, it provides directly actionable guidance for frontline clinical decision-making, which is precisely the value that distinguishes real-world research from RCTs and constitutes its irreplaceable societal impact.

Our results demonstrated that ICI-containing chemotherapy regimens significantly prolonged progression-free survival (PFS) compared with non-immunotherapy regimens, with a consistent favorable trend observed in overall survival (OS). Multivariable analysis further confirmed that ICI-containing chemotherapy regimens were independently associated with a reduced risk of disease progression. In this study, the survival curve of the chemo-ICIs group showed a separation trend in the early stage and maintained a stable advantage throughout the follow-up, suggesting that chemo-immunotherapy can not only delay disease progression, but also bring long-term survival benefits to patients. This result is highly consistent with the conclusions of many clinical studies in recent years (13, 23–28), further confirming the important role of chemo-immunotherapy in advanced NSCLC. It suggests that even in the real-world population without strict screening, this regimen can still maintain its survival benefit.

Given the promising efficacy observed, we next examined the safety profile of this regimen. Notably, the addition of ICIs to chemotherapy did not increase the overall incidence of adverse events. Certain hematologic and non-hematologic toxicities even exhibited lower rates in the non-ICI chemo group. This observation may be attributed to multiple factors. First, despite propensity score matching, clinicians tended to select patients with better baseline health status and organ function reserve for chemo-immunotherapy regimens. This selection bias—although partially controlled for observable covariates through PSM—cannot fully eliminate confounding by unmeasured factors. From a clinical practice perspective, immunotherapy combined with chemotherapy is currently the standard of care for patients with driver gene-negative NSCLC. Extensive studies have confirmed that this treatment strategy significantly prolongs survival. Importantly, the improved efficacy enables better overall tolerance and quality of life. Therefore, patients receiving chemo-immunotherapy regimens exhibited milder hematologic toxicity, fewer gastrointestinal reactions, and higher quality of life compared with those receiving chemotherapy alone. In contrast, patients receiving non-immunotherapy chemotherapy achieved inferior overall efficacy, and their performance status and quality of life tended to decline progressively with each treatment cycle, leading to a higher incidence of grade 3–4 adverse events requiring medical intervention. This may explain why the non-ICI chemo group paradoxically exhibited higher rates of severe adverse events in certain categories compared with the chemo-ICIs group. Second, more intensive monitoring in the chemo-ICIs group may have led to earlier detection and management of low-grade adverse events, thereby preventing their progression to severe events. Patients receiving chemo-immunotherapy typically undergo more frequent laboratory tests and imaging assessments, enabling timely intervention. Third, differential attribution of adverse events. Patients in the non-ICI chemo group, lacking the durable disease control effect of immunotherapy, experienced more rapid tumor progression and greater tumor burden. Tumor-related symptoms—such as cancer-related fatigue and cachexia—became more prominent in this group. In retrospective medical records, these symptoms may occasionally be difficult to distinguish from treatment-related adverse events, potentially inflating the recorded adverse event burden in the non-ICI chemo group. Prospective studies with larger sample sizes and standardized adverse event reporting are warranted to validate the safety profile of chemo-immunotherapy in real-world populations.

That said, the incidence of hypothyroidism was significantly higher in the chemo-ICIs group than in the non-ICI chemo group (13.5% vs. 4.5%), consistent with the well-established endocrine toxicity profile of immune checkpoint inhibitors (29, 30). In this study, the incidence of CIP was low (2.2% in the chemo-ICIs group, all grade 1–2), and no CIP cases were observed in the combination therapy group receiving anti-angiogenic agents. The reported incidence of CIP varies across studies. A systematic review and meta-analysis of 85 studies comprising 20,883 patients reported that the incidence of any-grade pneumonitis was 6% for PD-1 inhibitors and 8% for PD-L1 inhibitors, with grade ≥3 pneumonitis occurring in 3% and 2% of patients, respectively, and fatal pneumonitis (grade 5) in less than 1% of cases (31). The lower CIP incidence observed in our study compared with the reported rate of 6% for PD-1 inhibitors may be attributed to several factors, including the relatively limited sample size of this retrospective study, which may have affected the detection of rare events, as well as potential differences in the pneumonitis profile of domestic PD-1 inhibitors, the concomitant use of anti-angiogenic agents, and the absence of prior thoracic radiotherapy. In conclusion, the safety profile of chemo-immunotherapy in the real-world setting may be more favorable than anticipated, although prospective studies are warranted for further validation.

Among patients receiving chemo-immunotherapy, the addition of anti-angiogenic therapy also demonstrated a significant PFS benefit, whereas OS showed only a favorable trend without reaching statistical significance. When contextualizing our findings within the evidence-based framework of the combined immunotherapy, anti-angiogenic, and chemotherapy strategy, two landmark phase 3 trials serve as the primary reference points. The IMpower150 trial (32), an open-label phase 3 study, demonstrated that the addition of atezolizumab to bevacizumab plus carboplatin and paclitaxel (ABCP) significantly improved both progression-free survival (median 8.3 vs. 6.8 months; HR = 0.62) and overall survival (median 19.2 vs. 14.7 months; HR = 0.78) compared with bevacizumab plus chemotherapy alone (BCP) in patients with advanced non-squamous non-small cell lung cancer. However, the subsequent IMpower151 trial (33)—a randomized, double-blind phase 3 study specifically designed for the Chinese population, which enrolled 305 chemotherapy-naive Chinese patients with metastatic non-squamous NSCLC, of whom 97% received pemetrexed and 53% harbored EGFR mutations—failed to meet its primary endpoint of investigator-assessed progression-free survival (median 9.5 vs. 7.1 months; HR = 0.84; P = 0.184), with overall survival also showing no clinically meaningful improvement (median 20.7 vs. 18.7 months; HR = 0.93). Despite using the same immunotherapeutic and anti-angiogenic agents, these two trials yielded strikingly divergent results. The analytical logic of these two trials closely parallels that of our subgroup analysis. Both IMpower150 and IMpower151 assessed the value of adding ICIs to a chemo-anti-angiogenic backbone; in our study, we evaluated the addition of anti-angiogenic agents to a chemo-immunotherapy backbone. While not perfectly symmetrical, both approaches address the same central question: whether a third agent adds clinical benefit to a dual-therapy foundation. Our findings sit between the two—PFS benefit aligns with IMpower150, while the non-significant OS trend echoes IMpower151—illustrating the unique contribution of real-world evidence in reflecting heterogeneous patient populations and clinical practice.

The anti-angiogenic agents used in this study were predominantly recombinant human endostatin (Endostar) and bevacizumab. Although these agents differ in molecular structure and target specificity, they all exert similar immunomodulatory effects on the tumor microenvironment when combined with immunotherapy, primarily through inhibition of the VEGF/VEGFR pathway—promoting vascular normalization, alleviating tumor hypoxia, reducing myeloid-derived suppressor cells and regulatory T cells, and converting the “cold” tumor microenvironment into a “hot” one to enhance immune cell infiltration and function (34). Antiangiogenic drugs can improve the hypoxic state of the tumor microenvironment by remodeling tumor blood vessels to normalize them, so as to enhance the infiltration and function of immune cells. At the same time, immunotherapy can further inhibit tumor angiogenesis, and the two form a synergistic anti-tumor effect (35–37). The significant benefit of PFS in this study is the direct embodiment of this synergy. The benefit trend of OS may require longer follow-up or larger sample size for further verification.

Leveraging a broader and more heterogeneous patient cohort, this real-world investigation functions as a pragmatic stress test of evidence derived from idealized clinical settings. It exposes the potential limitations of directly extrapolating global trial results to distinct regional populations, while also underscoring the well-recognized challenge of translating PFS benefit into OS gain in the real-world setting. Importantly, it offers an authentic reflection of the complexities inherent in routine clinical care—complexities that arise from the coexistence of multiple comorbidities, heterogeneous patient characteristics, varied treatment histories, and the dynamic nature of clinical decision-making, all of which are systematically simplified or excluded in the tightly controlled environments of pivotal trials.

In addition, there was no significant difference in the incidence of AEs between the combined treatment group and the non-combined treatment group, suggesting that the combination of anti-angiogenesis therapy on the basis of immunotherapy did not significantly increase the toxicity and side effects. This result is consistent with the recent meta-analysis findings of Lin et al. (38), providing important safety evidence for the clinical application of combination therapy.

Of note, the data from 2018 to 2020 included in this study cover a unique transitional period in the clinical practice of chemo-immunotherapy for NSCLC in China. Although the pivotal KEYNOTE-189 (39) and KEYNOTE-407 (40) trials reported positive results in 2018, the adoption of chemo-immunotherapy as a widely accessible standard of care was gradual. The National Medical Products Administration (NMPA) approved pembrolizumab in combination with chemotherapy for first-line NSCLC in 2019. The Chinese Society of Clinical Oncology (CSCO) guidelines included the recommendation in 2019 and upgraded it to Category I in 2020. At the end of 2020, multiple PD-1 inhibitors were included in the National Reimbursement Drug List, and drug accessibility was substantially improved after the updated list took effect in 2021. Given that chemo-immunotherapy was in a transitional phase from approval to widespread adoption during 2018–2020, residual temporal confounding cannot be fully excluded despite the use of PSM to control for observable confounders. Any such residual bias is likely to be conservative.

This study has some limitations. Although this study is a multicenter study, only the case data of two medical institutions were included. Therefore, the extrapolation of research conclusions is limited. Moreover, the relatively small sample size, especially in subgroup analysis, may affect the statistical validity of the results. In addition, the data of AEs come from the retrospective extraction of medical records, and the records of AEs in clinical practice are mostly focused on serious events requiring clinical intervention, which can easily introduce bias in reporting. More importantly, although propensity score matching was employed to control for observable confounders, unmeasured baseline factors, such as nutritional status, frailty, and deeper organ function reserve, may still differ between groups. These factors are difficult to fully capture in retrospective studies but may influence patients’ tolerance to treatment and the risk of adverse events. Therefore, this safety finding should be interpreted with caution in this context. Another limitation is the lack of PD-L1 expression data. As PD-L1 expression is the most established predictive biomarker for immunotherapy, this missing information prevented us from performing subgroup analyses based on PD-L1 status and from adjusting for this key variable in the multivariate model, which may have limited the depth of our efficacy evaluation across different patient subsets. Finally, our results should be interpreted as a class-effect analysis of the combination strategy, and future large-scale studies are warranted to differentiate the efficacy and safety among individual agents.

In the future, we will conduct a multicenter, prospective cohort study, increase the sample size to enhance the statistical power, and further improve the internal validity and generalizability of the research results by rigorously controlling for baseline confounding factors. In addition, we can strive to optimize the criteria for molecular pathological stratification and identify the best beneficiaries of the triplet regimen (chemotherapy plus ICIs and anti-angiogenic agents), so as to more accurately evaluate the survival benefit and clinical application value of the combined regimen in the real world.

## 5 Conclusions

In unresectable stage III/IV NSCLC, the addition of ICIs to chemotherapy significantly improved PFS and OS compared with chemotherapy-based regimens without ICIs. Among patients receiving chemotherapy plus ICIs, adding anti-angiogenic agents provided further PFS benefit with acceptable toxicity but did not significantly improve OS. These findings support the integration of ICIs into chemotherapy backbones and suggest that anti-angiogenic agents may confer incremental PFS value, while their OS benefit requires further validation.

## Data Availability

The raw data supporting the conclusions of this article will be made available by the authors, without undue reservation.

## Supplementary material

Supplementary Table 1 Chemotherapy-based treatment regimens and regimen composition in the chemo-ICIs group and non-immunotherapy groups.

Supplementary Table 2 Distribution of specific immunotherapy and anti-angiogenic agents.
