# Supplementary material for: Efficacy and safety of chemotherapy plus immunotherapy in unresectable stage III–IV non-small cell lung cancer, with analysis of the additional role of anti-angiogenic agents
Source: Front Oncol. 2026 Aug 31;16:1897228. doi: 10.3389/fonc.2026.1897228 (PMC13573290; doi:10.3389/fonc.2026.1897228)
Supplement: Supplementary file 1 [file Table1.pdf]

Supplementary Table S1. Chemotherapy-based treatment regimens and regimen composition in the chemo-ICIs group and non-ICI chemo group.

Treatment Regimen Category	Specific Regimen	N
**Chemotherapy-based immunotherapy regimens (Chemo-ICIs)**	Chemotherapy + immunotherapy + anti-angiogenic therapy	71
	Chemotherapy + immunotherapy	91
**Non-immunotherapy chemotherapy-based regimens (Non-ICI chemo)**	Chemotherapy + anti-angiogenic therapy	48
	Chemotherapy alone	50

Supplementary Table S2. Distribution of specific immune checkpoint inhibitors and anti-angiogenic agents.

Drug Category/Specific Drug	N	Percentage within category (%)
**Immune Checkpoint Inhibitors (ICIs)**	162	
Tislelizumab	76	46.9%
Sintilimab	53	32.7%
Camrelizumab	31	19.1%
Toripalimab	1	0.6%
Pembrolizumab	1	0.6%
**Anti-angiogenic Agents**	119	
Recombinant Human Endostatin (Endostar)	71	59.6%
Bevacizumab	41	34.5%
Anlotinib	5	4.2%
Apatinib	2	1.7%
